# 

**Published:** 1888-05-19

**Authors:** 


Magistrate: "Have you no written document to prove that your wife is really dead?" Peasant: "I have the doctor's bill."—*Fliegende Blätter.*

A METHOD OF TESTING HUSBANDS.—A lady says: "If you want to find out a husband's real disposition take him when he's wet and hungry. If he's amiable then, dry him and fill him up, and you have an angel." There are husbands who decidedly object to the application of this test.

### Special Notice to Newsagents, Advertisers, and the Trade.

**CHANGE OF OFFICE AND PUBLISHER.**

Notice is hereby given that the Office of The Hospital will henceforth be at 38, Old Jewry (Cheap ide end), E.C., to which address all communications should be sent. Mr. A. Doig has been appointed Advertising Agent, and Mr. J. H. S. Hanning, Manager. All Cheques and Post Office Orders should be made payable to J. H. S. Hanning, 38, Old Jewry, E.C. crossed Lloyds, Barnetts, and Bosanquets Bank (Limited).

### To Secretaries and Matrons.

The Editor will be glad to have a copy of the Regulations, and also of every Report issued from the Press of Asylums, Hospitals, Conva'escent and Nursing Institutions, as well as those of other Charities, so soon as they are published, and an early copy in duplicate of all appeals for funds. Notices of Annual and other Meetings, Festival Dinners, etc., will be welcomed. All such communications should be addressed to *The Editor, The Lodge, Porchester Square, London, W.* Any superintendent, secretary, matron, or other chief official who will regularly send such documents and information will be registered to receive free of cost a cover for binding The Hospital in half-yearly volumes on sending name and address to the publisher.

### THE STUDENT'S COLUMN.

### EDINBURGH AND GLASGOW MEDICAL EXAMINATIONS.

THE quarterly examinations in Edinburgh of the Royal Colleges of Physicians and Surgeons, Edinburgh, and the Faculty of Physicians and Surgeons, Glasgow, for the triple qualification, took place in April and May with the following results :—

### FIRST EXAMINATION.

Of 62 candidates, the following 32 passed : Alfred Murray Gray, Isle of Man ; William Redmond Lemon County Armagh ; Miss Louisa Rosa Cooke, Yorkshire ; Miss Edith Mary Brown, Whitehaven ; Sidney Wood, Rochdale ; David Kidd Muir, West Hartlepool ; Charles Pearce, Chili ; Herbert Whalley, Bradford ; Fred. Hall, Lancashire ; Edmund Fergus Jamieson, County Down ; Daniel Doherty, County Donegal ; Paget Dundas Minchin, Kilkenny ; Rupert Wilberforce Clayton, Wrexham ; John Besnard Wilson, County Cork ; Edmund George MacSweeny, Macroom ; Andrew Hostage, Chester ; Patrick James Munn, Kirkcudbrightshire ; Thomas Marshall, South Shields ; Lawrence George Fink, Calcutta ; James Alden Fink, Calcutta ; Leslie Frank Bucknell, Sydney ; James Orr, County Antrim ; George Macdonald, Kirkintilloch ; Joseph Eugène Horace Gentil, Mauritius ; Richard Holmes, Westmeath ; Timothy White, Antrim ; John Charles Edwards, Llanrhaiadr ; Andrew Tannahill Brown, Dunfermline ; Robert Scott, Kinross-shire ; Daniel Vincent Ryan, County Cork ; Charles Edward Ross, Skye ; and Archibald Mackellar, Glasgow.

### SECOND EXAMINATION.

Of 83 candidates, the following 32 passed :—George Stevens Pope, Madras ; John Gordon M'Candlish, Leeds ; Henry Richard Preece, Cheltenham ; David Mark, Belfast ; Charles Pearse, Chili ; Samuel Wilson, Hanley ; William John Leitch, Tyrone ; Denis Michael Bernard Myers, Clonmel ; George Mason, Birmingham ; Herbert Henry Hodgetts, Addenbrooke, Smethwick ; Francis Horatio Amner, Kew ; Thomas Sprot Allan, Glasgow ; Wm Pirie, Arbroath ; George Milne Grieve, Dundee ; Robert Fawcitt Granger, Whitby ; Frederick Maurice Graham, Monmouth ; William Rae, Glasgow ; Patrick M'Elligott, Kerry ; Josue Marc Rohan, Mauritius ; William Redmond Lemon, County Armagh ; Walter John Haldane Cumming, Kent ; Edwin Andrew Cuthbert Hindmarsh, Calcutta ; John Nesbitt, County Derry ; Thomas Campbell Patterson, County Donegal ; Thomas Edgar Jones, South Wales ; Owen Wilmott Morgan, Ceylon ; John Stewart Boyd, Renfrewshire ; Archibald Robertson Douglas, Newcastle ; Thomas Young, Kilwinning ; Patrick Francis Godfrey, Tipperary ; William David Sweeny, County Mayo ; and Roderick Maclean, Ross-shire.

### FINAL EXAMINATION.

Of 70 candidates, the following 44 passed, and were admitted L.R.C.P.E., L.R.C.S.E., and L.F.P. and S.G. : Gilbert Alexander Bannatyne, Rutherglen ; Edward Archibald Simeon, Delhi ; Alfred Ellis Vaughan, Crewe ; William Alexander Frizell, Liverpool ; John Baldwin Meredith, Queen's County ; Sanderson Mellor, Huddersfield ; Robert Aldous, Norfolk ; Henry Buxton, Lancashire ; Frederick William Browning, Monmouthshire ; Andrew Blair Watson, Norfolk ; Frederick John Pacey, Melbourne ; William Yorke, Glasgow ; Cuthbert Ridley Pigg, Newcastle-on-Tyne ; James Knight Coutts, Aberdeen ; Kumar Bhabendra Narayan, Kuch Behar ; Albin Siddon, Bangalore ; Miss Jessie Crosfield, Liverpool ; William Grey, Northumberland ; Samuel James Dunlop, County Antrim ; Allan Mill, County Antrim ; Hitchon Chadwick, Burnley ; Albert Bradshaw, Sierra Leone ; Herbert Osborne, Notts ; Pranjivan Jazjivan Mehta, India ; Benjamin Evans Jones, Crewe ; James Hutcheson, Pestell, Australia ; Richard Francis Shaw, Hereford ; Cyril Herbert Sharpe, Norwich ; Charles Forrest Lovibond, Bridgewater ; William Bell, Enniskillen ; Alexander Macdonald, Edinburgh ; David Barry, County Cork ; William Walter Williamson, Scarborough ; Edward Ernest Craster, Middlesburgh ; Arthur George Laidler, Stockton-on-Tees ; Herman Fermor Lawrence, Tasmania ; Alfred Hunter Goodwyn, Devonshire ; George Oliver Moorhead, India ; Harry Goodsir Mackid, Canada ; Charles Smyth, Limerick ; Hardinge Charles Christian M'Niell, Folkestone ; George Herbert Douthwaite, Warminster ; Isaac Crawford, Omagh ; and Willam Beale Marston, junior, London.

During the April sittings of the Examiners of the Royal College of Surgeons, Edinburgh, Miss Jane Elizabeth Waterston, Inverness ; David Middleton Greig, Dundee ; and George Abbott, Titchmarsh, passed the final examination, and were admitted L.R.C.S.E. For the licence in dental surgery, Alfred Edward Donagan, Cambridge ; Edward John Montague Hodgkinson, London ; Thomas Gregory, Edinburgh ; John Stephen Walker, York ; Robert Keith-Common, Edinburgh ; Kevin Emmet O'Duffy, Dublin ; and Thomas Cuthill M'Kenzie, Edinburgh, passed the first professional examination ; and the following gentlemen passed the final examination, and were admitted L.D.S., Edinburgh :—George William Welham, London ; Herbert Bycroft Ezard, Bath ; John Stephen Walker, York ; and Kevin Emmet O'Duffy, Dublin.

THE following questions were given at the recent examinations for the triple qualifications of the Royal Colleges of Physicians and Surgeons of Edinburgh and the Faculty of Physicians and Surgeons of Glasgow :

### TRIPLE QUALIFICATION.

### FIRST EXAMINATION.

QUESTIONS IN CHEMISTRY (four questions, all of which are to be answered).—I. How can the compound nature of light be proved? II. Describe one method of preparing carbonic oxide gas, showing the equation. State its more important qualities. III. You are required to prepare 10 grains by weight of hydrogen, by acting on magnesium with dilute sulphuric acid : How many grains of each must be used? Give the equation. IV. Give the formula of ordinary starch, and indicate the chief sources from which it is obtained. What change is produced when starch is—(*a*) heated to between 210 deg. C and 280 deg. C ; (*b*) boiled with dilute sulphuric acid.

QUESTIONS IN ELEMENTARY ANATOMY (all of which are to be answered).—Give a brief description of the coccyx. II. Describe the posterior annular ligament of the wrist, and name in order the various tendons which pass under it. III. What movements take place at the ankle joint? Name the muscles by which they are effected. IV. Give the origin, insertion, and nerve supply of the biceps flexor cruris muscle.

### SECOND EXAMINATION.

QUESTIONS IN ADVANCED ANATOMY (all of which are to be answered). I. Describe the naked-eye anatomy of the medulla oblongata and pons varolii. II. Mention the naked-eye and microscopic characters by which you would distinguish between small and large intestine III. Describe the extrinsic muscles of the tongue ; and give their nerve supply and action. IV. Name the branches given off by the first part of the subclavian artery, and describe the distribution of the first branch.

QUESTIONS IN PHYSIOLOGY (all of which are to be answered.)-I. Describe the course of the fibres of the pyramidal tracts from the internal capsule downwards to the exit of the anterior nerve roots from the spinal cord. II. How and where is urea formed in the body? what is its chemical composition? how is it separated from the blood? and how much is excreted daily? III. Describe the manner in which the image of an external object is formed on the retina, and explain how "accommodation" for a near object is effected IV. Describe the movements of an ordinary respiration ; what are the muscles engaged? and what the nerves that supply them? Where is the respiratory nerve centre, and what are its afferent nerves?

QUESTIONS IN MATERIA MEDICA (three questions, all of which are to be answered).—I. Where is filix mas found? What part of the plant is used in medicine? How is the extract made, and what is its dose? II. To what order does gelsemium belong? Mention its preparations and doses. III. Give the composition of three mercurial ointments.

### FINAL EXAMINATION.

QUESTIONS IN THERAPEUTICS (all of which are to be answered, including the prescription).—I. How may the fœtor of breath in a case of bronchorrhœa be most effectively controlled, and the excessive secretion diminished. II. Mention some of the most approved remedies in the treatment of hæmoptysis and their mode of application. III. What are the most reliable indications for the use of stimulants in a case of continued fever? and how should they be prescribed? Prescription (to be written in unabbreviated Latin, the directions in English) : Prescribe an aperient containing calomel for a child of one year of age.

QUESTIONS IN MEDICINE (three only of which are to be answered).—I. Enumerate and describe the important signs and symptoms which you would expect to find present in a typical case of typhoid fever at the tenth day of the disease. Write out full instructions for the treatment of the case at this stage of the disease. II. Give the diagnosis of acute nephritis in the first stage of the disease ; the dangers to be apprehended ; and the treatment by which these might be averted. III. Describe the characteristic abnormal muscular movements in each of the following diseases : Paralysis agitans, multiple cerebro-spinal sclerosis, chorea, locomotor ataxia, athetosis. IV. Enumerate the skin diseases included in the class neuroses Give the pathology, symptoms, course, and treatment of herpes zoster affecting the trunk.

QUESTIONS IN SURGERY (all of which are to be answered).—I. Give (1) the symptoms of dislocation of the lower jaw, (2) the method of reduction. II. Describe (1) the different varieties of bursitis, and (2) the treatment suitable to each. III. Tetanus—(1) What are its causes? (2) Distinguish it from strychnia poisoning, and from hydrophobia. IV. Polypus of the nose—(1) What are the varieties? (2) What sites do they respectively occupy? (3) Give the treatment of each.

SURGICAL ANATOMY (one of which only is to be answered).—I. Describe the relations of the peritoneum to the ascending, transverse, and descending colon. II. Trace the course of the internal pudic artery, and mention its branches.

QUESTIONS IN MIDWIFERY AND GYNÆCOLOGY (three questions must be answered, of which the last must be one).—1. Extra uterine pregnancy :—its classification and causes. State how its diagnosis is to be attempted? II. Into how many stages is labour divisible? Describe what occurs in each, and in normal labour in a primipara, their relative duration. III. Should a woman suffering from uterine fibroid be allowed to marry? What may be the consequences to her if she marry? IV. Give the symptoms and physical signs of hæmatocele, the treatment at onset, and also subsequently.

QUESTIONS IN MEDICAL JURISPRUDENCE AND HYGIENE (four questions, of which three are to be answered, and in these 3 and 4 must be included).—I. What are the actions of carbolic acid? What is a poisonous dose, the symptoms produced by it, and the treatment? What post-mortem appearances may be found when it has been taken in excess by mouth? II. What is meant by fœticide? Mention some of the methods of producing criminal abortion ; the penalty accruing for effecting or trying to effect the same ; and what would be the steps you would take if requested to examine and report upon a suspected case. III. Mention the most important salts of lead from a toxicological point of view. Describe the symptoms of chronic lead poisoning, mentioning two ways by which it may accidentally gain access to the system, and how to prevent their occurrence. Give two tests for lead. IV. What is meant by density of population, and how is it usually expressed? What would you consider the death rate indicating a good sanitary condition of a town to be ; and what condition of the population, in regard to age, may have a marked effect upon this rate?

### Amusements with Profit for All Ages.

**Rules.**—All answers must be addressed to the Prize Editor, The Hospital, 38, Old Jewry, Cheapside, London, E.C., not later than the *first* post on Thursday next. The full name and address must in all cases be given, but a *nom de plume* may be added for publication. Only one side of the paper must be written on.

### FOR MEN AND WOMEN.

**Rule.**—Competitors can enter for *all* quarterly competitions, but no competitor may take more than *two quarterly prizes* during the year.

### Acrostic Competition.

Second quarter commenced February 25th, 1888; ends May 19th, 1888.

A PRIZE of ONE GUINEA will be given to the competitor who gains the highest number of marks for best original acrostics during the ensuing quarter. All acrostics sent in for this competition will be credited according to merit, twelve marks being the highest score given for one acrostic.

A PRIZE of HALF-A-GUINEA to be given to the competitor who gains the highest score for solution of acrostics set. *One mark* only will be given to the competitors who solve all the lights of each acrostic.

Exception will be made in the case of *quotation acrostics*, when *two marks* will be given, *one* for the correct solution of all *lights*, and *one* mark for the *source* of the quotations.

This week credit will be given for solution of

No. 7 Double Acrostic.

"One of these men is genius to the other;
. . . . Which is the natural man?
And which the spirit? Who deciphers them?"

1. "A king of the Saxons, ere yet his last he breath'd,
To the merry Monks of Croyland his drinking-horn bequeath'd."

2. . . . . "And one may drink, depart,
And yet partake no venom."

3. "Mother of spleen, in robes of various dye,
Who vexéd was full oft with ugly fit;
And some her frantic deem'd, and some her deem'd a wit."

4. "Where smiling spring its earliest visit paid,
And parting summer's lingering blooms delay'd."

5. "His cup was vanish'd, for in secret guise
The younger guest purloin'd the glittering prize."

6. "I could lie down like a tired child
And weep away the life of care."

7. "Where hang in equipoise
The night and day."

8. "It will not, will not rest! Poor creature, can it be
That 'tis thy mother's heart which is working so in thee?"

. . . . "Now we clap
Our hands, and cry!"

10. "In hope to merit heaven by making earth a hell."

11. "Now God us shield, it was revealed,
That he his bed must make;
Within a tomb, and share its gloom,
With a black and living snake."

12. "Thought would destroy their paradise."

### Answers.

No. 6. Double Acrostic.

W arrio R
A rgyl E
L egen D
T odri G
E v A
R oussea U
S aladi N
C our T
O 'Connel L
T hul E
T ournamen T.

Correct answers have been received from Oliver, Reldas, Dunce, Lady Clara, Brisbane.

### THE WORD COMPETITION.

Third Quarterly Competition commenced May 5th, 1888; ends July 28th, 1888.

Three prizes of one guinea, 15*s.* and 10*s.* 6*d.* will be given each quarter to the three competitors who obtain the highest number of marks by making the largest number of words derived from the word or phrase set for anatomical dissection; that for this, the *third* week of the quarter, being

"Cricket."

Observe.—Proper names, abbreviations, foreign words, words of less than four letters, and repetitions are barred; plurals, and past and present participles, of verbs are allowed. Nuttall's *Standard* dictionary only to be used.

N.B.—All words sent in must be arranged *alphabetically*, with correct total affixed.

### Results of First Week.

*Broad Arrow.*—Dollie (33), Tinie (33), Reldas (32), Brisbane (32), Patience (32), Lightowlers (32), Lauriston (32), Pec (31), Sym (31), Carmen (30), Dunce (29), Geranium (28), Puff (26), Robe (25), Oliver (25), M. R. (25).

### Puzzles—Third Week.

No 9 Charade.

My *first* is the short of a feminine name;
The French as a pronoun my *second* may claim;
My *whole* is familiar to all as a fish,
And is highly esteemed as a savoury dish.

No. 10. Square Word.

. . . . 1. A bird.

. . . . 2. A flower.

. . . . 3. A fabled mountain.

. . . . 4. Prosperity.

No. 11. Double Acrostic.

If in the world you wish to rise,
Either of these a way supplies.

1. Invade these haunts I would not dare.
2. A state of mind that makes you stare.
3. "Queen and huntress, chaste and fair."
4. Author of many a pretty air.
5. In fields oft bearded, men's are bare.
6. A painter fine of pictures rare.

No. 12. Enigma.

Two brothers wisely kept apart,
Together ne'er employed;
Though to no purpose we are bent,
Each takes a different side.
We travel much, yet pris'ners are,
And close confined to boot,
Can with the swiftest horse keep pace,
Yet always go on foot.

### Results of First Week Puzzles.

No. 1. Riddle-me-ree.—Crab.

No. 2. Decapitations.—Smart, Mart, Art.

No. 3. Geographical Charade.—Con-naught = Connaught.

No 4. Double Acrostic.

C hesterfiel D
H awa I
A driati C
R yswic K
L ucern E
E islebe N
S evre S

*All right.*—Mount Edgecumbe, Spider, Nunkey, Scrooge, Geranium, Judy, Alsie, Happy Eliza, A. C. K., Kelpie, McCulloch, The Young Canadian, Inanity, Plummy Fluff, Hercules. *Three right.*—Baby, Sambo, G. H. R. H.

### Conditions.

I. Every paper sent in must be clearly written, and one side only must be used. All competitors must mark at the head of their papers the number of their competition.

II. Each paper must be signed by the author with his or her real name and address. A *nom de plume* may be added, if the writer does not desire to be referred to by us by his real name. In the case of all prizewinners, however, the real name and address will be published.

III. All communications relating to prize competitions should be addressed to "The Prize Editor, The Hospital, 38, Old Jewry, London, E.C." Competitors are requested not to include in letters, etc., to the Prize Editor communications on matters of other business. All letters must be fully prepaid.

IV. The Prize Editor of The Hospital is the sole judge of the competitions, and his decision is in all cases final and without appeal.

V. The Prize Editor reserves the right to publish any paper sent in, whether it receives a prize or not. He does not hold himself responsible for any MSS. sent, nor can he undertake to return rejected competitions.

VI.—All children resident at school are requested to forward their *home* address in addition to the school one, when entering for the "Children's Corner" Competitions.

### A CHINESE DOCTOR AND HIS FEES.

The new district magistrate of Shanghai has taken the native doctors in hand. Lately, he sent one of his messengers to a well-known doctor, with a fee of 600 cash—about 60 cents.—to ask him to visit a patient. As the messenger had strict orders not to say he came from the magistrate, the doctor was under the impression that the patient was not an official, and accordingly refused to go. Again the messenger was sent, and again the doctor refused to attend, saying the fee was too small, and that he would not go for three times the amount. The third time the magistrate sent his own card, and the doctor at once hastened to see him. On being interrogated why he had not come in the first instance he made various excuses, which the magistrate cut short by observing that in future he would cut down the doctor's fees to such a low figure that it would not be worth his while to continue practising. He gave the doctor the alternative of paying 5,000 taels (6,000 dols.) to the Yellow River Fund. The fine was ultimately reduced to 3,000 taels, and the doctor, it is recorded, was very glad to get off so easily.

**Notice to all Correspondents.**—N.B.—All letters referring to this page, which do not arrive at 38, Old Jewry, Cheapside, London, E.C., by the *first post on Thursdays*, and are not addressed PRIZE EDITOR, will in future be disqualified and disregarded.

No one will be permitted to win the Monthly Prizes twice in any one year, the Annual prize being offered especially to prevent this.

